# Ergonomics in the operation-theatre: a healthcare provider-based cross-sectional study

**DOI:** 10.1097/MS9.0000000000001538

**Published:** 2023-12-02

**Authors:** Oshan Shrestha, Sunil Basukala, Niranjan Thapa, Sagun Karki, Lochan Shrestha, Melina Shrestha, Bipin Kumar Mehta, Bikesh Raj Sunuwar, Pujan Maharjan

**Affiliations:** a College of Medicine; b Department of Surgery, Nepalese Army Institute of Health Sciences, Kathmandu, Nepal

**Keywords:** Ergonomics, Operating Room, Surgery

## Abstract

**Background:**

Performing surgery is a task that demands mental stability, precision, and vigilant eyes, along with resilient physical strength, as surgeons and those who assist the surgeons have to assume a sustained, difficult posture that can go on for hours. About 23–100% of surgeons report musculoskeletal discomfort that originates from poor ergonomics.

**Methods:**

Ethical clearance for the study was obtained. This cross-sectional study, conducted in a tertiary centre among the healthcare providers working inside the operating room, spanned from 1 March 2023, to 26 June 2023. Systematic sampling was applied, and consent was obtained before data collection. A structured questionnaire was used as the study tool, and the collected data was analysed in SPSS 20.

**Results:**

A total of 98 personnel responded, among which 67.3% were males and 32.7% were females, with a median age of 36 (32–42) years. Only 6.1% of the workers had received training on ergonomics. The prevalence of work-related musculoskeletal disorders was 82.7%, and more than two-thirds of the participant’s life outside of work was affected by this. More than two-thirds (69.4%) felt their work environment was not safe, and surgeons performing open surgery were at lower odds of feeling that their work environment was safe.

**Conclusion:**

There is a high prevalence of work-related musculoskeletal disorders among healthcare providers working inside the operating room, and the majority had their body position deviated from neutral most of the time during the surgery. There is a deficiency in ergonomic practices, which demands an effective intervention.

## Introduction

Highlights

- About 6% of the healthcare workers had only received training on ergonomics.
- The majority had their body position deviated from neutral most of the time during the surgery.
- The prevalence of self-reported work-related musculoskeletal disorders was 82.7%.
- More than two-thirds felt their work environment was not safe for their physical health.
- Identified barriers were lack of training on ergonomics, uncooperative co-workers, and a substandard working environment.

The act of performing surgery is a task that not only demands mental stability, precision, and vigilant eyes, but it also demands resilient physical strength, as surgeons and those who assist the surgeons have to assume sustained difficult posture that can last for hours. Also, surgery includes the use of various instruments, including sharp blades, which puts the surgeons at risk of injury. This makes the application of ergonomic principles in the operating room very important. It is reported that between 23 and 100% of the surgeons report musculoskeletal discomfort that originates from poor ergonomics^1^. The basic concept of ergonomics is building an environment that suits the worker instead of forcing workers to fit into the work environment^2^. Lack of adequate discussion on ergonomics, training on ergonomics, and its lack of application put the surgeon and assistants at greater risk of experiencing musculoskeletal discomfort. This affects the surgical speed, concentration of the surgeon, precision in the work life, sleep, and quality of life in the personal life^3^.

This study has taken surgeons and those who assist during surgery into focus to study how their work environment has affected them. This study aims to study the knowledge of ergonomic principles and their applications, posture during surgery, work-related musculoskeletal disorders, and barriers to achieving ergonomic work practices.

## Methods

This study is in line with STROCSS reporting guidelines^4^.

### Protocol registration

The protocol followed in this study was registered in the Research Registry.

### Study design and setting

This cross-sectional study was conducted in a tertiary-level referral centre among the healthcare providers (intern doctors, residents, registrars, consultants, and surgical technicians) working inside the operating room. This study, conducted with the theme of exploring ergonomics in operating theatre, was conducted from 1 March 2023, to 26 June 2023. Healthcare providers working under the surgery and orthopaedics disciplines during the study period and giving consent to participate in the study were included in the study, while any deviation from the inclusion criteria was not entertained. Written informed consent was obtained before the data collection, and the participants were assured of confidentiality.

### Sample Size and sampling technique

For sample size calculation we used Cochran’s formula considering the heterogenous and large population. Details of sample size calculation is available as Supplementary File 1, Supplemental Digital Content 1, http://links.lww.com/MS9/A319.


$$\begin{array}{c} n=Z^{2}\times \left(p\times q\right)/e^{2}\\ \;=1.96^{2}\times \left(0.642\times 0.36\right)/0.05^{2}\\ \;=355 \end{array}$$

where,

*n* = calculated sample size

*Z* = 1.96 at 95% CI

*P* = expected work-related musculoskeletal disorder among healthcare providers working in the operation room, 64.2%^5^

*q* = 1−*p*

*e* = Margin of error (5%)

Total number of healthcare providers in Surgery and Orthopaedics department during the study period (*N*): 130


Adjustedsamplesize(n’)=n/(1+n/N)=355/(1+355/130)=95

Considering 5% non-response rate, the final sample size was 100.

Systematic sampling method was used for the selection of the participants. A list of healthcare providers (intern doctors, residents, registrars, consultants, and surgical technicians) was prepared in alphabetical order, and every fourth person (a computer-generated random number) on the list was selected as a participant in the study.

### Study tool

A structured questionnaire was formulated for data collection that collected data on different headings like demographic details (age, sex, height, department, majority type of surgery performed, years in the field, and surgeries performed per week), knowledge and application of principles of ergonomics, posture while performing surgery, work-related musculoskeletal disorder, satisfaction scale, and barriers. The study tool was formulated after a literature review and adaptation of principles of ergonomics, recommendations for good posture during surgery, and the Nordic musculoskeletal questionnaire. The questionnaire was face-validated after peer review. The study tool was then pretested and reformed according to the feedback received. Details of the study tool are available in Supplementary File 1, Supplemental Digital Content 1, http://links.lww.com/MS9/A319.

### Variables

All of the dependent variables (DV) and independent variables (IV) of this study were categorical except for age, height, years in the field, and surgeries performed in a week, which were continuous variables. The DV of this study was work-related musculoskeletal disorder (WSMD) and the 5-point Likert item for satisfaction scale. The WSMD variable is treated as nominal, while the 5-point Likert item for the satisfaction scale is treated as an ordinal variable^6^.

### Analytical strategy

Frequency was calculated for all the categorical DV and IV of the study. The Shapiro–Wilk test was used to check the normality of the data. For continuous variables, mean with SD or median with inter-quartile range (IQR) were used as a measure of central tendency and dispersion for normal and skewed data, respectively. The prevalence of WSMD was calculated with a 95% CI, and binomial regression was used for WSMD (no WSMD coded as 0 and presence of WSMD coded as 1) to study the relationship between it and IV of the study. For the 5-point Likert item, while mentioning the frequency of positive responses, strongly agree and agree were combined, and while mentioning the frequency of negative responses, disagree and strongly disagree were combined. The Mann–Whitney U test (as IV had two categories) was used to see if any difference existed between the categories of categorical IV and responses to the Likert item. Statistically significant categorical variables and Likert items were then treated with ordinal regression with other continuous IV as co-variates.

## Results

Out of 100, a total of 98 personnel responded and were part of this study. Among the 98 participants, 67.3% were males and 32.7% were females, with a median (IQR) age of 36 (32–42) years. The median (IQR) height of the participants was 1.62 (1.55–1.68) metres, and their years in the field were 5 (3–9) years. The participants performed a median of 10 (6.75–13) surgeries per week. Demographic details of the participants are given in Table 1.

**Table 1:** Demographic details of the participants.

	Sample group (*N*= 98)
Items	*n* (%)
Age (in years)	
24–29	14 (14.28)
30–39	48 (48.97)
40–49	34 (34.69)
50 and above	2 (2.04)
Sex	
Male	66 (67.3)
Female	32 (32.7)
Specialty	
Orthopaedics	44 (44.9)
Surgery	54 (55.1)
Majority surgery performed	
Open surgery	71 (72.4)
Minimal invasive surgery	27 (27.6)

This study found that only 6.1% of the participants had received training on ergonomics, and only 15.3% of the healthcare providers knew about the principles of ergonomics. The majority of the participants avoided working in awkward body positions (86.7%), adjusted table height (89.8%), avoided unnecessary actions during surgery (88.8%), and worked under a good lighting source (88.8%), but only 31.6% took breaks to relax and stretch in between long hours of surgery. Healthcare providers that felt principles of ergonomics were not practically applicable due to a substandard working environment were 90.8%, and those who felt principles of ergonomics were not practically applicable due to uncooperative co-workers were 55.1%. The details of responses to the question regarding knowledge and application of ergonomic principles are given in Table 2.

**Table 2:** Related to knowledge and application of ergonomic principles.

	Sample group (*N*= 98)
Items	*n* (%)
Received training on ergonomics	
Yes	6 (6.1)
No	92 (93.9)
Know about the principles of ergonomics	
Yes	15 (15.3)
No	83 (84.7)
Avoid working in awkward body positions	
Yes	85 (86.7)
No	13 (13.3)
Always have all the equipment within easy reach during surgery	
Yes	53 (54.1)
No	45 (45.9)
Adjust working height and put minimal strain on back and neck	
Yes	88 (89.8)
No	10 (10.2)
Avoid unnecessary actions during the surgery	
Yes	87 (88.8)
No	11 (11.2)
Take breaks to relax and stretch in between long hours of surgery	
Yes	31 (31.6)
No	67 (68.4)
Provision of good lighting source	
Yes	87 (88.8)
No	11 (11.2)
Instruments function optimally	
Yes	72 (73.5)
No	26 (26.5)
Personal protective gears are well-functioning	
Yes	71 (72.4)
No	27 (27.6)
Principles of ergonomics cannot be followed due to substandard working environment	
Yes	89 (90.8)
No	9 (9.2)
Principles of ergonomics cannot be followed due to uncooperative co-workers	
Yes	54 (55.1)
No	44 (44.9)

During the surgical procedures, 61.2% of the participants reported that their heads were tilted by more than 15°, and 51.0% had their backs flexed by more than 10°. The majority of the participants shifted weight occasionally from one leg to another (83.7%), kept hands at the level between the waist and middle of the chest (77.6%), and their arms remained in the position of reaching forward by less than 18 inches (69.4%). Details of the responses related to posture during surgery are given in Table 3.

**Table 3:** Related to the posture during surgery.

	Sample group (*N*= 98)
Items	*n* (%)
Most of the time during work, my head remains	
Vertical	11 (11.2)
Tilted less than 15°	27 (27.6)
Tilted more than 15°	60 (61.2)
Most of the time during work, my back remains	
Upright	9 (9.2)
Flexed less than 10°	39 (39.8)
Flexed more than 10°	50 (51.0)
Most of the time during work, my arm remains	
Hung by side of the body	5 (5.1)
Reaching forwards less than 18 inches	68 (69.4)
Reaching forward more than 18 inches	24 (24.5)
Most of the time during work, my hands are positioned at height	
Above the centre of the chest	19 (19.4)
Between waist and middle of the chest	76 (77.6)
Below the level of the waist	3 (3.1)
Most of the time during work, I	
I lock both knees and stand straight	7 (7.1)
Shift weight occasionally from one leg to another	82 (83.7)
Bear weight in one leg most of the time	9 (9.2)

The point prevalence of self-reported work-related musculoskeletal disorder (WSMD) was found to be 82.7% (95% CI: 75.5–89.8), and the most commonly affected region was the lower limbs, followed by the lumbar and cervical regions (Figure A, Supplementary File 2, Supplemental Digital Content 2, http://links.lww.com/MS9/A320). This study found that more than two-thirds of the participant’s work life and life outside of work are affected by this. A feeling of discomfort while performing surgical procedures was felt most of the time by 64.3% of the healthcare providers. Details of the responses to the work-related musculoskeletal disorder are given in Table 4.

**Table 4:** Related to the work-related musculoskeletal disorder.

	Sample group (*N*= 98)
Items	*n* (%)
Are you experiencing musculoskeletal discomfort due to work?	
Yes	81 (82.7)
No	17 (17.3)
How often do you experience significant discomfort while performing surgery?	
3 or more times a week (most of the times)	63 (64.3)
1–2 times a week (sometimes)	32 (32.7)
Less than once a week (insignificant)	3 (3.1)
How has this affected your work life?	
No difference in performance	4 (4.1)
Somewhat affected my performance	39 (39.8)
I cannot work properly like I used to	55 (56.1)
How has this affected your daily life outside of work?	
Not affected at all	5 (5.1)
Cannot exercise, socialize or go outside due to fatigue/pain	29 (29.6)
Cannot even perform household chores due to fatigue/pain	64 (65.3)

On running the Pearson chi-square test of association between categorical IV and WSMD, it showed no significant association between the variables (Table A, Supplementary File 2, Supplemental Digital Content 2, http://links.lww.com/MS9/A320). Univariate binomial regression was performed for WSMD and continuous IV. The dependent variable (WSMD) had no significant relationship with the continuous IV. The details of the regression analysis are given in Table 5.

**Table 5:** Details of the binomial regression analysis.

							95% CI	
Variables	Coeff	S.E.	Wald	df	*P*	OR	Lower	Upper
Age	-0.048	0.04	1.38	1	0.24	0.95	0.88	1.03
Height	0.87	3.67	0.05	1	0.81	2.41	0.002	3210.4
Years in field	-0.05	0.05	0.93	1	0.33	0.94	0.85	1.05
Surgeries performed per week	-0.06	0.05	1.23	1	0.26	0.93	0.83	1.05

Coeff, coefficient; df, degrees of freedom; OR, odds ratio; SE, standard error.

When the healthcare providers were asked if they felt their work environment was safe for their physical health, more than two-thirds (69.4%) felt their work environment was not safe, while 9.2% agreed that their work environment was safe for their physical health. Details of the 5-point Likert item are given in Table 6.

**Table 6:** 5-point Likert item for satisfaction scale.

Likert item	Strongly disagree, *n* (%)	Disagree, *n* (%)	Neutral, *n* (%)	Agree, *n* (%)	Strongly agree, *n* (%)
I feel my work environment is safe for my physical health	15 (15.3)	53 (54.1)	21 (21.4)	8 (8.2)	1 (1.0)

Mann–Whitney U test was used to see if any difference existed between the responses to the Likert item and categorical IV. It showed that the responses had a statistically significant difference among the 5-point Likert item and major type of surgery performed variables (U= 724.50, *P* value= 0.041) (Table B, Supplementary File 2, Supplemental Digital Content 2, http://links.lww.com/MS9/A320). Other variables did not show significant results. Statistically significant variable was then treated with ordinal regression along with continuous IV as co-variates. Ordinal regression showed that surgeons performing open surgery were at lower odds of feeling their work environment to be safe (odds ratio= 0.348, *P* value= 0.031). Details of the analyses are available in Table 7.

**Table 7:** Details of ordinal regression.

							95% CI	
Variables	Coeff	S.E.	Wald	df	*P*	OR	Lower	Upper
Age	0.034	0.07	0.203	1	0.65	1.03	0.891	1.202
Height	2.149	2.74	0.611	1	0.43	8.57	0.03	1877.12
Years in field	-0.042	0.10	0.159	1	0.69	0.95	0.77	1.18
Surgeries performed per week	-0.01	0.05	0.068	1	0.79	0.98	0.89	1.08
Major type of surgery performed								
Open	-1.05	0.48	4.641	1	0.03	0.34	0.13	0.90
Minimal invasive	Reference							

Coeff, coefficient; df, degrees of freedom; OR, odds ratio; SE, standard error.

The barriers felt by the participants towards the application of ergonomic principles were most felt at the policy level of the institution (48.0%), followed by at the level of the executive body (27.6%). About 5% of the participants felt that the barrier lied at the level of the operating surgeon. Details of the responses are given in Table C, Supplementary File 2, Supplemental Digital Content 2, http://links.lww.com/MS9/A320.

## Discussion

Surgeons are often compared with athletes on the grounds of their leadership, teamwork, complex body movements, long hours of training, mindfulness, and visualisation^7,8^. This points out the importance of ergonomics in the operating room even more for safe and efficient operation, assistance, and communication. This study found that only 6.1% of the respondents had received training on ergonomics, and 15.3% of the respondents were aware of the principles of ergonomics. While 89.8% of the participants adjusted their working height, putting minimal strain on their back and neck, 88.8% avoided unnecessary actions during surgery, and 31.6% took breaks in between long hours of surgery. Looking at the number of participants receiving training on ergonomics, the percentage of participants adjusting table height, avoiding unnecessary actions, and taking breaks in between is high. However, this is still low and can be raised significantly after training the healthcare workers. This has been demonstrated by Franasiak *et al*.^9^ in their study, where the self-reported level of pain decreased and the implementation of healthy practices increased with surgical ergonomic training.

The neutral body position while operating has components like the neck not getting tilted more than 15°, the back being flexed less than 10°, the hand reach not exceeding 18 inches, the hands being positioned in between the waist and middle of the chest, and shifting weight occasionally from one leg to another^10^. This study assessed the posture of healthcare workers working in the operating room and found that most of the time during surgery, 61.2% had their neck tilted by more than 15°, 51.0% had their back flexed by more than 10°, 24.5% had their hands reaching by more than 18 inches, 22.5% did not have their hands positioned between the waist and middle of the chest, and 16.3% did not shift weight occasionally from one leg to another. This puts the healthcare worker at greater risk of developing musculoskeletal disorders. The self-reported prevalence of WSMD was found to be 82.7% (95% CI: 75.5–89.8). The fact that the prevalence of WSMD among health workers working in the operating room remains high across the world is shown by the various studies. Yizengaw *et al*.^5^ found 64.2%, Owada *et al*.^11^ showed 65.1%, Vaghela *et al*.^12^ showed 83.70%, and Rață *et al*.^13^ found 95.78% prevalence. The findings of this study are consistent with these results. The lower limbs, lumbar region, and cervical region were the top three most affected body parts in this study. Also, Tavakkol and colleagues, in their meta-analysis with the inclusion of 12 studies, showed that these regions were mostly affected. This issue needs immediate intervention, as the respondents to this study reported that they could not exercise, socialise, or go outside due to fatigue or pain, while the majority reported that they could not even perform household chores due to fatigue or pain. On running the Pearson chi-square test and regression analysis, the presence of WSMD was not found to be significantly associated with the other variables of the study. The burden of WSMD was high and was not associated with any specific variable. Respondents to this study were asked if they felt their work environment was safe for their physical health, and only 9.2% agreed to this, and more than two-thirds did not agree to this. Regression analysis showed that surgeons performing open surgery more frequently had lower odds of feeling their work environment was safe. This may be associated with the need to stand for long hours, the direct handling of sharp instruments, and more exposure to bodily fluids.

More than 90.8% of the respondents reported that the principles of ergonomics cannot be followed due to a substandard working environment, and 55.1% reported that they cannot be followed due to uncooperative co-workers. About 75% of the respondents perceive that the barrier to achieving an ergonomic work environment in their setting lies at the policy level or executive body level. This has demanded a policy-level change to reduce the burden of WSMD. Also, the issue of uncooperative co-workers can be solved by establishing a healthy work culture and providing training on ergonomic practices.

This study is not without limitations. This study was a single-centric study, taking only the Surgery and Orthopaedics department into account and not taking other surgical specialties into account. Also, the study tool used in this study was an adaptation of a validated tool, not the tool itself. The study tool was face-validated but not statistically validated. This leaves the question of the generalizability and accuracy of the study findings open. However, the data obtained from this study can act as the basis for further specific, goal-directed studies for intervention to reduce the burden of work-related musculoskeletal disorders among healthcare professionals working inside operating rooms and to promote an ergonomically sound working environment.

## Conclusion

There is a high prevalence of work-related musculoskeletal disorders among healthcare providers working inside the operating room, and the majority had their body position deviated from neutral most of the time during the surgery. There is a deficiency in ergonomic practices, which demands an effective intervention. The identified barriers, like the low percentage of workers receiving training and knowing about ergonomic principles, the substandard working environment, and uncooperative co-workers, need policy-level intervention from the stakeholders.

## Ethical approval

Ethical clearance for this study was received from Institutional Review Committee of Nepalese Army Institute of Health Sciences (Ref no: 798).

## Consent

Written informed consent was taken before the data collection and is available from the corresponding author on reasonable request.

## Source of funding

This article did not receive any grants.

## Author contribution

O.S. and S.B., and N.T.) were involved in conceptualization of the study. O.S., N.T., S.K., L.S., and M.S. were involved in taking informed consent and data curation. O.S. and N.T. did the formal analysis. O.S., S.K., B.K.M., B.R.S., and P.M. were involved in initial manuscript drafting. S.B., L.S., and M.S. revised the manuscript from intellectual aspect. All the authors proofread and approved the final version of the manuscript.

## Conflicts of interest disclosure

There are no conflicts of interest.

## Clinical trial registration

N/A.

## Guarantor

Oshan Shrestha, MBBS, Nepalese Army Institute of Health Sciences, Kathmandu, Nepal E-mail: shresthaoshan93@gmail.com.

Sunil Basukala, MBBS, MDHA, MS, Nepalese Army Institute of Health Sciences, Kathmandu, Nepal E-mail: sunil.basukala@naihs.edu.np.

## Data availability

Collected data that were analysed are available from the corresponding author on reasonable request.

## Provenance and peer review

Not commissioned, externally peer-reviewed.

## Supplementary Material

SUPPLEMENTARY MATERIAL
