# Supplementary material for: Ergonomics in the operation-theatre: a healthcare provider-based cross-sectional study
Source: Ann Med Surg (Lond). 2023 Dec 2;86(1):127–32. doi: 10.1097/MS9.0000000000001538 (PMC10783321; doi:10.1097/MS9.0000000000001538)
Supplement: SUPPLEMENTARY MATERIAL [file ms9-86-127-s001.docx]

**Study tool**

**Demographic details:**

Age:

Sex:

Height:

Specialty (eg. Ortho):

Majority type of surgery performed (Open/ minimal invasive):

Time of being involved in surgical field (in years):

Number of surgeries performed/assisted per week (in average):

**Related to knowledge of principles of ergonomics and its application:**

1. Have you ever been trained on ergonomics? Y/ N
2. Do you know about the principles of ergonomics? Y/ N
3. I avoid working in awkward body positions. (other than neutral body position): Y/ N
4. I always have all the equipment within easy reach during surgery: Y/ N
5. I adjust working height and put minimal strain on my back and neck. Y/ N
6. I avoid unnecessary actions during the surgery: Y/ N
7. I take breaks to relax and stretch in between long hours of surgery: Y/ N
8. There is provision of good lighting source: Y/ N
9. All the instruments function optimally: Y/ N
10. All of the personal protective gears (like lead jacket) are of standard quality? Y/ N
11. Even though I am made aware about the ergonomics, I cannot follow it due to substandard working environment. Y/ N
12. Even though I am made aware about the ergonomics, I cannot follow it due to uncooperative co-workers. Y/ N
13. Most of the time during work, I:
14. I lock both knees and stand straight
15. Shift weight occasionally from one leg to another
16. Bear weight in one leg most of the time

**Related to posture during performing surgery:**

1. Most of the time during work, my head remains:
2. Vertical
3. Tilted less than 15 degrees
4. Tilted more than 15 degrees.
5. Most of the time during work, my back remains:
6. Upright
7. Flexed less than 10 degrees
8. Flexed more than 10 degrees
9. Most of the time during work, my arm remains:
10. Hung by side of the body
11. Reaching forwards less than 18 inches
12. Reaching forward more than 18 inches
13. Most of the time during work, my hands are positioned at height:
14. Above the centre of the chest
15. Between waist and middle of the chest
16. Below the level of the waist

**Related to work related musculoskeletal disorder (WSMD):**

1. Are you experiencing musculoskeletal discomfort due to work?
2. Yes
3. No
4. Which region is more affected? (check all that apply)
5. Cervical Pain
6. Lumbar Pain
7. Shoulder Pain
8. Arm Pain
9. Leg Pain
10. How often do you experience significant discomfort while performing surgery?
11. 3 or more times a week (most of the times)
12. 1 to 2 times a week (sometimes)
13. Less than once a week (insignificant)
14. How has this affected your work life?
15. No difference in performance
16. Somewhat affected my performance
17. I cannot work properly like I used to
18. How has this affected your daily life outside of work?
19. Not affected at all
20. Cannot exercise, socialize or go out with friends due to fatigue/pain
21. Cannot even perform household chores due to fatigue/pain

**Likert scale: (please tick)**

I feel my work environment is safe for my physical health? [Strongly Dis; Dis; Neutral; Agree; Strongly Agree]

**Barriers:**

Where is the barrier towards achieving ergonomic work place in your setting? (Select one that most applies according to you)

1. Fault in the policy level that runs the hospital
2. At the level of the executive body/administration
3. At the level of the department itself
4. At the level of the operating surgeon
5. At the level of the OT incharge
