# Supplementary material for: Ergonomics in the operation-theatre: a healthcare provider-based cross-sectional study
Source: Ann Med Surg (Lond). 2023 Dec 2;86(1):127–32. doi: 10.1097/MS9.0000000000001538 (PMC10783321; doi:10.1097/MS9.0000000000001538)
Supplement: SUPPLEMENTARY MATERIAL [file ms9-86-127-s002.docx]

**Table A.** Pearson Chi-Square test of association

| **Analyses** | **Pearson Chi-Square** | **df** | **p-value** |
| --- | --- | --- | --- |
| **WSMD * Sex** | 0.098 | 1 | 0.754 |
| **WSMD * Specialty** | 0.039 | 1 | 0.844 |
| **WSMD * Major type of surgery performed** | 0.036 | 1 | 0.850 |

*WSMD: Work-related musculoskeletal disorder, df: degrees of freedom*

**Table B.** Mann-Whitney U test

| **Analyses** | **Mann-Whitney U** | **Z score** | **p-value** |
| --- | --- | --- | --- |
| **I feel my work environment is safe for my physical health* Sex** | 985.50 | -0.587 | 0.557 |
| **I feel my work environment is safe for my physical health* Specialty** | 1165.00 | -0.181 | 0.857 |
| **I feel my work environment is safe for my physical health* Major type of surgery performed** | 724.50 | -2.045 | 0.041 |

**Table C.** Perceived barrier level

| **Items** | **Sample group (N= 98)** | |
| --- | --- | --- |
|  | **n** | **%** |
| **Barrier towards achieving ergonomic work place in my setting lies at:** | | |
| **At the policy level that runs the institution** | 47 | 48.0 |
| **At the level of the executive body/administration** | 27 | 27.6 |
| **At the level of the department itself** | 15 | 15.3 |
| **At the level of the OT In-charge** | 4 | 4.1 |
| **At the level of the operating surgeon** | 5 | 5.1 |

**Figure A.** Most affected regions
